# Biomarkers of heavy metals pollution in mangrove ecosystems: Comparative assessment in industrial impact and conservation zones

**DOI:** 10.1016/j.toxrep.2025.102011

**Published:** 2025-03-25

**Authors:** Rozirwan, Nadila Nur Khotimah, Wike Ayu Eka Putri, Fauziyah, Riris Aryawati, Gusti Diansyah, Redho Yoga Nugroho

**Affiliations:** a Department of Marine Science, Faculty of Mathematics and Natural Sciences, Universitas Sriwijaya, Indralaya, South Sumatra 30862, Indonesia; b Environmental Management Study Program, Graduate Program, Universitas Sriwijaya, Palembang 30139, Indonesia

**Keywords:** Biomarkers, Conservation zones, Heavy metals, Industrial activities, Mangrove

## Abstract

Heavy metal contamination from industrial activities in coastal regions can lead to pollution in mangrove ecosystems. Mangroves produce antioxidant compounds to mitigate the impact of free radicals. This study aimed to analyze the correlation between the concentration of heavy metals Pb and Cu and antioxidant activity in *Avicennia alba* and *Excoecaria agallocha* mangroves from areas affected by industrial activities and conservation areas, Banyuasin, South Sumatra, Indonesia. This study was conducted in September 2023 with sampling locations in the Payung Island area and the Barong River conservation area, Berbak Sembilang National Park. The samples taken included sediment and mangrove leaves. The concentration of heavy metals Pb and Cu was measured by atomic absorption spectrometry. Antioxidant activity test using the DPPH test, total phenol using the Folin-Ciocalteu method, and phytochemical profile screening using GCMS. Statistical analysis of the correlation between antioxidant activity and heavy metal concentration using the Pearson correlation. The results showed that the highest concentration of heavy metals in sediment and mangrove leaves was found in the area affected by industrial activity, with a range of Pb values of 0.67 ± 0.16–18.70 ± 0.48 mg/kg and Cu values of 3.39 ± 0.20–6.07 ± 0.37 mg / kg. The results of sediment pollution assessment for heavy metals Pb and Cu at Igeo < 0 indicates uncontaminated, 1 < Cf < 3 indicates low contamination, and PLI 0–2 indicates not polluted. While the results of heavy metal bioaccumulation in leaves were BCF < 1, indicates low bioaccumulation. *E. agallocha* leaves from the Pulau Payung area showed very strong antioxidant activity of 21.63 μg/ml, and the highest total phenol content reached 398.80 mg GAE/g. Analysis of compounds with the highest antioxidant activity identified the presence of esters, aldehydes, alcohols, fatty acids, glycosides, flavonoids, terpenoids, and steroids. Correlation analysis shows that higher heavy metal concentrations correspond to increased antioxidant activity and total phenol content (r ≠ 0). These findings are expected to contribute to scientific knowledge that enhances environmental sustainability, supporting effective management of coastal natural resources.

## Graphical Abstract

**Figure:**
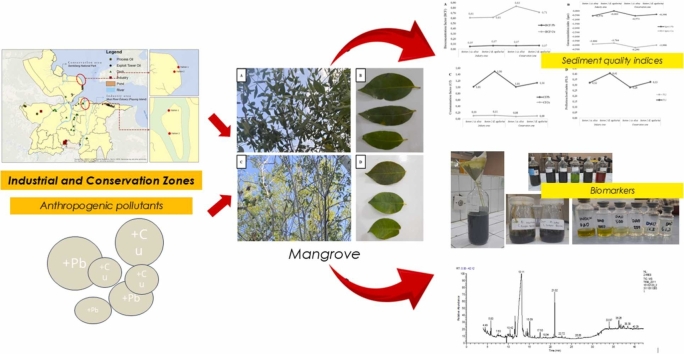

## 1 Introduction

Coastal areas are transitional areas between land and sea that have abundant biodiversity and unique ecosystems [1], [2]. Coastal areas face great pressure from various anthropogenic activities that can cause pollution [3], [4]. Previous studies report that industrial activities like fertilizer processing, oil and gas, and crude palm oil production contribute to coastal pollution [3], [5], [6]. In addition, there are also agricultural activities, ports, shipping, loading and unloading of coal raw materials and their products, and households [7]. Continuous anthropogenic activities in coastal areas can produce pollutants, such as microplastics, heavy metals, as well as various organic and inorganic contaminants [8], [9], [10]. Among various pollutant types, heavy metals are categorized as persistent pollutants due to their resistance to decomposition [11]. Heavy metals initially present in the water column gradually settle to the sediment and eventually accumulate in aquatic organisms [12]. This condition may have adverse impacts, particularly if it exceeds environmental quality standards. These adverse impacts can affect aquatic ecosystems, including mangroves [13], [14]. According to Xu et al., [15], as the largest plant community in coastal areas, mangroves are also directly affected by pollution.

Mangrove ecosystems play a vital role in coastal protection, supporting biological diversity, and contributing to the socio-economic development of local communities [16], [17]. Additionally, their capacity to accumulate pollutants makes them valuable indicators for assessing pollution levels in coastal waters, as they can absorb and store these pollutants in their tissues, enhancing their role in monitoring environmental health [18], [19], [20]. Roots and leaves are important parts of mangroves in the absorption, accumulation, and response to pollutants [21]. Roots are the first part exposed to pollutants from their growth media. Furthermore, roots also have the ability to translocate pollutants to the leaves. Leaves are the primary site for photosynthesis in plants, supplying the energy essential for cell development, and overall plant function [22]. High concentrations of pollutants in roots and leaves can potentially increase excessive reactive oxygen species (ROS), resulting in oxidative stress in mangroves [23], [24]. Oxidative stress arises from an imbalance between ROS production and detoxification, potentially leading to harmful cellular damage [25], [26]. Although oxidative stress can be detrimental, plants also have a resistance response mechanism against free radicals [27]. This process involves producing antioxidant enzymes and molecules to counteract the harmful effects of free radicals. In response to environmental changes, plants enhance the activity of antioxidant defenses, including both enzymatic and non-enzymatic components such as superoxide dismutase (SOD), catalase (CAT), peroxidase (POD), glutathione peroxidase (GPx), and phenolic compounds. These antioxidants serve as protective mechanisms against various environmental stress [28], [29].

Research on the specific physiological adaptations of various mangrove species to pollutants is still limited. Most previous studies only focused on the accumulation of heavy metals in mangroves without exploring in depth the biochemical defense mechanisms they employ [30], [31], [32]. However, studies on how different mangrove species respond to industrial pollution in environments with varying levels of pollution have not yet been conducted. In addition, most studies only examine one mangrove species without comparing the adaptability of different species in the face of heavy metal contamination [33], [34].

This study aimed to evaluate the accumulation of heavy metals (Pb and Cu) in two mangrove species (*Avicennia alba* and *Excoecaria agallocha*) and assess their antioxidant activity in industrial and conservation zones. The selection of these two species was based on their prevalence in the research location as well as differences in habitat zones and morphological characteristics [35], [36]. This study was carried out in the mangrove ecosystem, which includes areas influenced by industrial activities such as Payung Island as well as conservation areas in the Berbak Sembilang National Park [37], [38].

By assessing biomarkers, new insights are provided into how mangrove species adapt to environmental stress caused by heavy metal pollution. Additionally, the research explores the impact of heavy metal contamination on the physiological responses of mangroves, focusing on their biochemical defense mechanisms. The findings aim to enhance understanding of mangrove adaptation strategies in response to pollution and offer valuable implications for coastal ecosystem conservation and environmental pollution management.

## 2 Materials and method

### 2.1 Leaf sampling

**Fig. 1:**
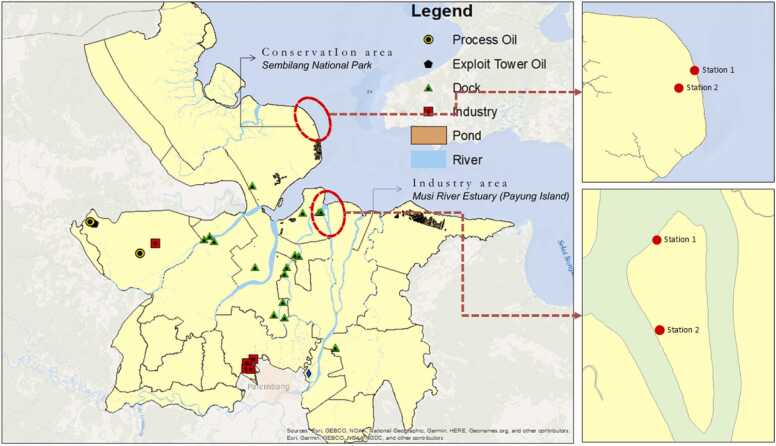
Map of sample collection.

This study was conducted in September 2023. The samples included *Avicennia alba*, *Excoecaria agallocha*, and sediments collected from industrial and conservation zones in Banyuasin, South Sumatra, Indonesia (Fig. 1). The first area is the mangrove ecosystem on Payung Island. This area was chosen due to the high accumulation of heavy metals from industrial activities along the Musi River. Additionally, the area includes agricultural activities, ports, fish ponds, and settlements [39], [40]. The second area is the Barong River conservation area, Berbak Sembilang National Park, which represents a natural area and protects flora and fauna from the threat of damage, scarcity, or deforestation [41], [42], [43].

The sampling stages include collecting sediment samples and mangrove leaves. Sediment samples were taken as supporting data to determine the concentration of heavy metals in the mangrove growth media. The availability of heavy metals in sediments has a direct effect on the bioaccumulation and biomagnification processes in aquatic organisms. Sediment data helps understand the level of risk and potential impacts to organisms in mangrove ecosystems. Sediment samples were taken using a grab pipe at a depth of ± 10 cm from the surface [44]. Sediment depth shows a very significant impact on heavy metal content, with a greater decrease in heavy metal content as sediment depth increases [45]. Samples were taken at three location points for each station, which were considered as replications. Samples were taken compositely together (taken as needed, 500 g) and placed into a polyethylene plastic container and stored in a cool box for analysis in the laboratory.

The method for collecting mangrove leaves taken from the field uses a random sampling method [46]. The random sampling method can be used if the sample studied is homogeneous. The mangrove species taken were *A. alba* and *E. agallocha*. The samples taken consisted of ± 1 kg of leaves and were put in polyethylene plastic.

### 2.2 Sediment grain size analysis

Grain size analysis was conducted using the sieving and pipetting methods as outlined by [47]. Sediment types (gravel, sand, silt, and clay) were classified using Shepard's triangle analysis and processed with Microsoft Excel V.2021, following the protocols established by [48], [49]. The sediment fraction type was determined by identifying the most dominant composition from the analysis results.

### 2.3 Sample preparation

Sediment sample preparation involved removing foreign objects such as plastic fragments and leaves. The sediment was then air dried at room temperature for 72 hours until fully dry, ground to a homogeneous consistency, and stored in a tightly sealed polyethylene bottle. They were then air-dried in a shaded, well-ventilated area for five days, ensuring indirect exposure to sunlight to prevent the degradation of bioactive compounds. The drying process was conducted at ambient temperature with sufficient airflow to facilitate moisture evaporation. Once dried, the samples were ground into a fine powder and stored in sealed containers for further analysis. The extraction of heavy metals (Pb and Cu) from the sediment samples and mangrove leaves was performed using the wet destruction method, following the procedures outlined by [8], [50].

### 2.4 Atomic absorption spectroscopic measurement

Measuring the concentration of heavy metals Pb and Cu using an Atomic Absorption Spectrophotometer (Shimadzu AA-7000). Operational parameters: Pb (283.3 nm, 5 mA lamp current) and Cu (324.7 nm, 4 mA), slit width 0.5 nm, air-acetylene flame (2.0 L/min air; 1.5 L/min acetylene), burner height 5–7 mm. After 15–20 min warm-up, calibration was performed using blank and standard solutions (0.1–2.0 ppm Pb; 0.05–1.0 ppm Cu), achieving R² ≥ 0.995. Samples were aspirated in triplicate with 15-sec distilled water rinsing between measurements and acid blank checks every 5 samples. Quality control included spike recovery (85–115 %), duplicate analyses (RSD <5 %). LODs: 0.02 ppm Pb; 0.01 ppm Cu (3 ×SD blank) [51].

### 2.5 Determination of heavy metals in leaves and sediments

#### 2.5.1 Determination of sediment pollution

##### 2.5.1.1 Geoacumulation index (I_*geo*_)

The Igeo (geo-accumulation index) quantitatively evaluates the degree of heavy metal contamination and classifies the level of pollution based on detailed categorization [52].

(1) Igeo = log_2_ (Cn/1.5 Bn)

The classification of Igeo values includes the following categories: uncontaminated (Igeo ≤ 0), uncontaminated to moderately contaminated (Igeo 0–1), moderately contaminated (Igeo 1–2), moderately to highly contaminated (Igeo 2–3), highly contaminated (Igeo 3–4), highly contaminated to very highly contaminated (Igeo 4–5), and very highly contaminated (Igeo ≥ 5) [53].

### 2.6 Contamination factor (Cf)

The contamination factor is determined experimentally as the ratio of the element concentration in the sample to its background concentration [54].

(2) Cf = (Cn/Bn)

The contamination factor (Cf) classifications are as follows: [55]: Cf < 1 = low contamination; 1 < Cf < 3 = moderate contamination; 3 < Cf < 6 = sufficient contamination; Cf > 6 = very high contamination.

### 2.7 Pollution load index (PLI)

The pollution load index (PLI) is utilized to assess pollution quality in a given area. The pollution load index value uses the formula [56].

(3) PLI = [*Cf1 x Cf2 x Cf3…. x Cfn*] ^1/n^

Pollution load index (PLI) criteria: PLI 8–10 = severely polluted; PLI 4–8 = heavily polluted; PLI 2–4 = moderately polluted; PLI 0–2 = not polluted to lightly polluted; PLI < 0 = not polluted.

### 2.8 Bioaccumulation of metal in leaves

#### 2.8.1 Bioconcentration factor (BCF)

The absorption of metals by leaf from sediment occurs through a process known as bioaccumulation. The bioconcentration factor (BCF) values are utilized to assess the extent of metal bioaccumulation in mangrove leaf originating from sediment [57].

(4) BCF = (Cn.leaf/Cn. sediment)

BCF > 1 hyperaccumulator; BCF = 1 indicator; BCF < 1 is an excluder [58].

### 2.9 Analysis of antioxidant non-enzymes in leaves

#### 2.9.1 Antioxidant activity evaluated by DPPH assay

Antioxidant activity analysis was carried out using ethanol solvent based on a method adapted from [59]. A 50 ml 0.1 μM DPPH solution was prepared, followed by the preparation of a sample stock solution and a 10 ml pure ascorbic acid stock solution of 2000 ppm, which was homogenized. Furthermore, a series of solutions were made with concentrations of 1000 ppm, 500 ppm, 250 ppm, 125 ppm, and 62.5 ppm. At each concentration, 1 ml of 0.1 μM DPPH solution was added to the mixture, which was then homogenized and incubated in the dark for 30 minutes. After incubation, the absorbance was measured using a UV-Vis spectrophotometer (Shimadzu UV-1900, Japan) at a wavelength of 517 nm. The antioxidant activity of the extract is expressed as IC_50_, which quantifies the strength of its antioxidant capacity (Table 1). The IC_50_ value is calculated using the following formula:

(5) $$\%\mathrm{inhibition}=\;\frac{\mathrm{blank abs}-\mathrm{sample abs}.}{\mathrm{blank abs}}x100\%$$

**Table 1:** Characteristic value of IC_50_.

Concentration (μg/ml)	Characteristic
< 50	Very strong
50–100	Strong
100–150	Moderate
150–200	Low

The IC_50_ value was derived by inputting the data into a linear regression equation, where the sample concentration was plotted on the X-axis and the percentage of inhibition of antioxidant activity on the Y-axis. The regression equation used is represented as y = ax + b [60].

### 2.10 Determination of phenol content

The analysis of total phenol content in the samples was conducted using the Folin-Ciocalteu method, as outlined in the literature [60], [61], [62]. A standard solution of 1000 ppm gallic acid as much as 50 ml was prepared, then variations in concentrations of 10 ppm, 20 ppm, 30 ppm, 40 ppm, and 50 ppm were made, each as much as 5 ml. For each concentration variation, 1 ml, 2 ml, 3 ml, 4 ml, and 5 ml were pipetted into a 10 ml measuring flask containing a standard solution of 100 ppm gallic acid. A total of 50 mg of sample was weighed, then 2 ml of methanol and 5 ml of distilled water were added, then homogenized in a 10 ml measuring flask. In both the standard series and sample variations, 0.5 ml of 50 % Folin-Ciocalteu reagent was added, followed by the addition of distilled water up to the mark. The mixture was then allowed to stand for 5 minutes. Next, one ml of a 5 % Na_2_CO_3_ solution was added and incubated in a dark place for one hour. After incubation, the absorbance of the sample was measured using a UV-Vis spectrophotometer at a wavelength of 750 nm.

### 2.11 Pearson correlation analysis (*correlation bivariate*)

The use of pearson correlation analysis (bivariate correlation) is a method used to evaluate the relationship between two variables [63], [64], in this case to see the relationship between antioxidant activity and heavy metal concentrations. This analysis was carried out using SPSS software version 28.

## 3 Result and discussion

### 3.1 Description of mangrove leaves

**Fig. 2:**
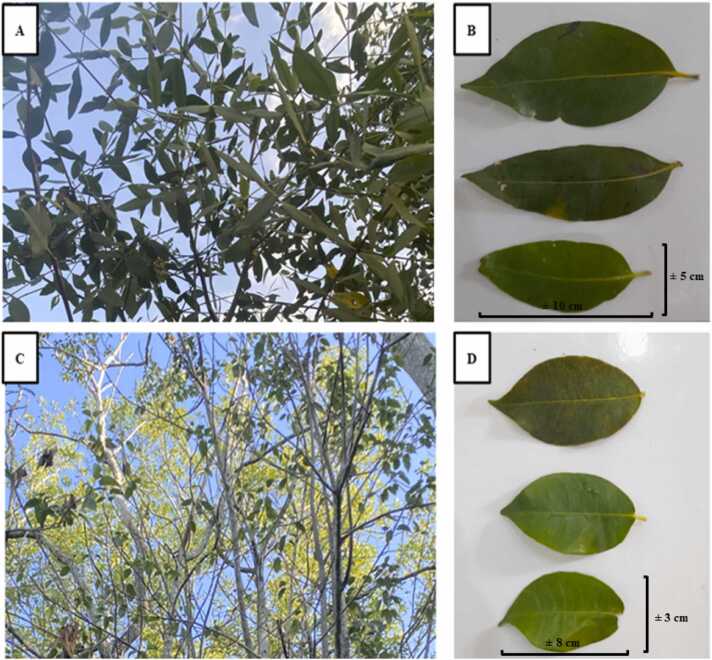
Leaves description. A-B). *A. alba*, C-D). *E. agallocha*.

The mangrove species *A. alba* and *E. agallocha* found at the sampling location exhibit distinct characteristics. Fig. 2 shows the characteristic differences between *A. alba* and *E. agallocha* leaves.

Leaves are the part that characterizes a mangrove species. When identifying each type of mangrove, observation of the morphology of the leaf shape is very important to understand the characteristics and differences in each type of leaf [66], [65]. *A. alba* leaves have a green surface with a smooth and slippery texture, while the underside is yellowish green with a rough texture. The characteristics of the leaves is elliptical, almost oval, with a tapered tip. Based on observations, the length of the leaves ranges from 10 to 13 cm, and the width of the leaves ranges from 4 to 5 cm. *E. agallocha* leaves are elliptical and dark green in color, with finely serrated edges and tapered tips. The observed leaf sizes ranged from 8 to 10 cm in length and 3–4.5 cm in width. Old leaves were selected as samples for the study of heavy metal content and bioactive compounds due to several considerations related to their maturity and potential accumulation of pollutants and compounds of interest. According to [67], plants tend to produce bioactive compounds in higher amounts in older parts. This could be a plant strategy to protect itself from pests, diseases, or the external environment [68], [69]. Older leaves may have more stable chemical conditions, thus facilitating analysis and minimizing variability in results.

### 3.2 Sediment grain size

**Fig. 3:**
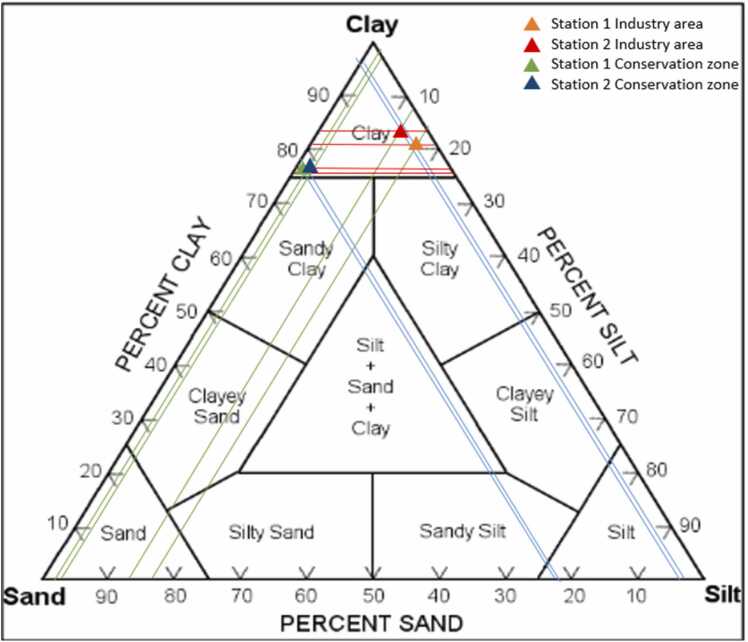
Classifications of sediment type with shepard triangle method.

The determination of substrate types in the sampling was conducted using the Shepard triangle method (Fig. 3). In the mangrove ecosystem of both industrial and conservation areas, sediment substrates were categorized into four types: gravel, sand, mud, and clay. The results indicated that the predominant substrate type in both areas was clay.

The sediment substrate surrounding the mangrove ecosystem in both areas is predominantly clay, with clay percentages ranging from 80.5 % to 84.03 %. The highest clay content was observed at station 1 in the industrial area (Table 2).

**Table 2:** Sediment grain size in each station.

Location	Station	Sediment fraction (%)				Grain size
		Gravel	Sand	Mud	Clay	
Industry area	1 (*A. alba*)	0.00	3.6	12.37	84.03	Clay
	2 (*E. agallocha*)	0.00	3.36	16.14	80.5	Clay
Conservation zone	1 (*A. alba*)	0.00	22.5	1.95	75.55	Clay
	2 (*E. agallocha*)	0.00	21.91	2.02	76.07	Clay

Based on the results of Table 2, distribution of sediment fractions and grain sizes at two different locations, which represents two stations with different mangrove species *(A. alba* and *E. agallocha*). In the industrial area, most of the sediments consist of clay with a very low sand content (3.6 % for Station 1 and 3.36 % for Station 2), which indicates the predominance of fine materials that can influence the mobility of heavy metals and nutrients in the sediments. In contrast, in the conservation zone, although the sediment composition is still dominated by clay, the sand content is higher (22.5 % for Station 1 and 21.91 % for Station 2), indicating differences in sedimentation processes and a higher potential for water infiltration.

In the industrial area, both stations (*A. alba* and *E. agallocha*) showed a dominance of clay fractions with a very high percentage. The dominant clay fractions indicate that the sediment in this area consists of fine particles, which may be caused by the accumulation of fine particles from industrial activities around this location. Industries such as fertilizer processing, oil and gas, crude palm oil production, agricultural activities, ports, shipping, loading and unloading of coal raw materials and their products, and households contribute to the presence of fine particles in sediments [3], [5], [6], [7]. Port activities involve frequent vessel movement, dredging, and cargo handling, all of which can resuspend fine particles and increase sedimentation rates [70], [71]. Crude oil processing and petroleum industries may contribute to fine particle deposition through air emissions, which settle via atmospheric deposition [72]. Additionally, agricultural activities, particularly palm oil plantations, can contribute to increased fine particle accumulation through soil erosion and runoff carrying clay-rich sediments into adjacent water systems, particularly during heavy rainfall [73], [74].

Fine particles such as clay are usually carried by water and can accumulate in areas with slow water movement, such as near mangrove roots [75], [76]. In the conservation zone, the clay fraction also dominates, although with a slightly lower percentage than the industrial area. The conservation zone may also have less influence from human activities, so the sediment pattern is more natural than the industrial area. Clay is a sediment particle with a very fine grain size and a large surface area [77], [78]. Due to its small size and its tendency to be negatively charged, clay has a high adsorption capacity, which allows clay particles to attract and bind heavy metal ions such as Hg, Pb, Cd, Cu, and others [79], [80], [81]. Consequently, sediments dominated by clay fractions tend to accumulate more heavy metals than larger sediment fractions [82], [83].

### 3.3 Determination of heavy metals

The results of the heavy metal concentration analysis for Pb and Cu in sediments and mangrove leaves from both areas are summarized in Table 3. The concentrations of heavy metals in sediments from both the industrial area and the conservation zone exhibit variability; however, they generally remain below hazardous thresholds (ERL, ERM, TEL, and PEL). In the industrial area, the highest Pb concentration was found at Station 2 (18.70 ± 0.48 mg/kg), while in the conservation zone, the highest concentrations of Pb and Cu were each at Station 2 (Pb 14.22 ± 0.16 mg/kg; Cu 5.17 ± 0.17 mg/kg). For metal accumulation in mangrove leaves, Cu was recorded higher than Pb at all stations. In the industrial area, *A. alba* (Station 1) had Pb 0.67 ± 0.17 mg/kg and Cu 3.39 ± 0.20 mg/kg, while *E. agallocha* (Station 2) showed Pb 1.27 ± 0.31 mg/kg and Cu 3.73 ± 0.16 mg/kg. In the conservation zone, the highest accumulation of Cu in mangrove leaves was 3.69 ± 0.23 mg/kg at Station 2.

**Table 3:** Average concentrations of heavy metals (mg/kg) in mangrove sediments and leaves.

	Pb	Cu
Sediments		
Station.1 Industry area	12.63 ± 0.01	5.58 ± 0.05
Station.2 Industry area	18.70 ± 0.48	6.07 ± 0.37
Station.1 Conservation zone	12.61 ± 0.32	4.21 ± 0.03
Station.2 Conservation zone	14.22 ± 0.16	5.17 ± 0.17
ERL	46.7	34
ERM	218	270
TEL	30.2	18.7
PEL	112	108.2
Mangrove leaves		
Station.1 Industry area (*A. alba*)	0.67 ± 0.17	3.39 ± 0.20
Station.2 Industry area (*E. agallocha*)	1.27 ± 0.31	3.73 ± 0.16
Station.1 Conservation zone (*A. alba*)	0.84 ± 0.12	3.50 ± 0.35
Station.2 Conservation zone (*E. agallocha*)	0.99 ± 0.37	3.69 ± 0.23

The industrially impacted area in the Musi River Estuary is affected by high anthropogenic activities, making it susceptible to accumulating pollutants, especially heavy metals such as Pb and Cu. Sediments in this area tend to contain higher pollutants than water and biota, influenced by domestic, industrial, and river transportation activities that pollute the environment [84], [85]. Ship and coastal building maintenance activities, including the use of anti-rust materials, electronic waste, and pipe corrosion, are the main sources of Pb, while sources of Cu in the aquatic environment come from antifouling paint, agricultural pesticides, and industrial waste [86], [87]. In addition, previous studies report that cleaning ship hulls can release Cu into the marine environment [88], [89]. Fisheries sector that uses Cu-coated nets to prevent biofouling can also contribute to increasing Cu levels in waters [90], [91].

The conservation area in the Barong River is also exposed to heavy metal pollution, although at a lower level, considering that some human activities such as fishing are still ongoing [92]. Unmanaged anthropogenic activities, including unregulated industrial waste disposal, improper wastewater treatment, and uncontrolled agricultural runoff, have contributed to the increasing Cu concentrations observed in both locations, as indicated by the findings of this study and previous research [7], [50]. These activities introduce Cu into the aquatic system, where it binds to suspended particles and accumulates in sediments, further exacerbating environmental pollution.

In mangrove leaves, Pb was detected at low concentrations. Plants regulate Pb primarily by limiting its uptake and translocation. Because Pb is a non-essential and highly toxic metal, most of it accumulates in the roots rather than being transported to the leaves [93]. In contrast, Cu an important micronutrient for plants, is regulated through controlled absorption and detoxification mechanisms [93], [94]. Plants manage excess Cu by binding it to metallothionein and phytochelatin, storing it in vacuoles, and activating the antioxidant defense system to fight oxidative stress [95], [96]. Although heavy metal concentrations vary between locations, they are still below the threshold, indicating a relatively low risk of contamination. However, long-term monitoring is essential to track bioaccumulation trends in mangrove ecosystems.

### 3.4 Sediment quality indices

**Fig. 4:**
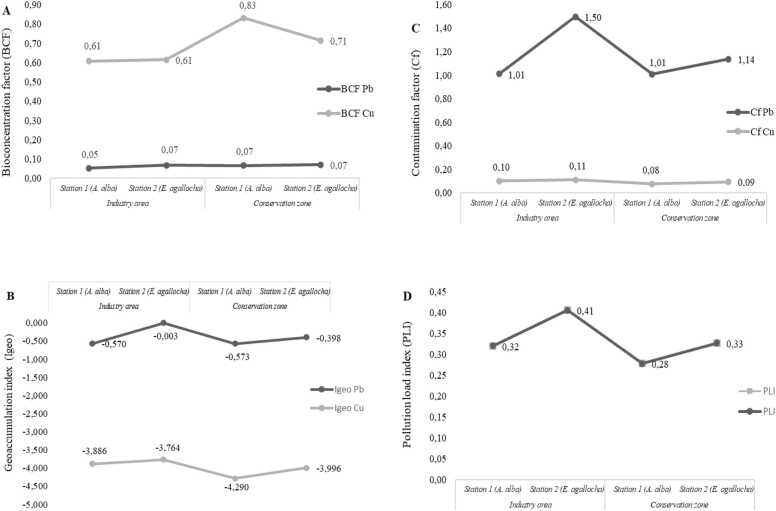
Sediment quality indices. A). Bioconcentration factor (BCF), B). Geoaccumulation index (Igeo), C). Contamination factor (Cf), and D). Pollution load index (PLI).

The results of the sediment quality index assessment are summarized in Fig. 4. The results of the leaf bioconcentration factor (BCF) in bioaccumulating Pb and Cu metals from sediment with a BCF value < 1 indicating low bioaccumulation. The geoaccumulation index shows uncontaminated properties for Pb and Cu with an Igeo value < 0 indicating uncontaminated. The contamination factor (Cf) shows that contamination is low and moderate in Pb and Cu with a value of 1 <Cf < 3 indicating low contamination. The PLI ranges from 0 to 2 indicating that both areas are not polluted.

The difference in bioconcentration factor (BCF) between Cu and Pb can be explained by the chemical properties of each metal. Cu accumulates more easily in biota tissues than Pb. Cu is an essential element for organisms, although at higher concentrations it can be toxic [97]. In contrast, Pb is a non-essential heavy metal that tends not to accumulate much in biota tissues [98]. The previous studies stated that essential heavy metals are more easily absorbed by organisms because they have physiological mechanisms to regulate the concentration of these elements [98], [99]. Analuddin et al. [100] have also examined BCF in mangrove ecosystems, with results showing that the BCF values for Hg, Cu, Mn, Pb, and Zn > 1. This finding is thought to be related to the impact of anthropogenic activities in Kendari City, which has a high population density.

The Igeo index shows higher Pb contamination than Cu in industrial areas. Pb is thought to originate from human activities such as ports, agriculture, ship transportation, and household waste that tends to settle in sediments [32], [79]. A high Igeo value indicate an anthropogenic contamination and show sediments contaminated heavy metal [101]. In addition, long-term exposure to these heavy metals can change community structure and disrupt ecosystem function through bioaccumulation and biomagnification in the food chain [102], [103].

The high value of the Pb contamination factor (Cf) in industrial areas indicates that this environment is more susceptible to Pb pollution than Cu. Relevan study by Hasan et al. [104] that the CF value of Pb (0.76) > Cu (0.68) in core sediment from a mangrove at the Pasur River. Cu is more likely to be bound to organic particles and accumulate in the tissues of benthic organisms, which may explain the lower Cf Cu value. The areas suspected of being polluted tend to have higher anthropogenic activity than conservation zones, which causes significant differences in the levels of contamination and accumulation of heavy metals [105]. Industries around mangrove areas may contribute to elevated levels of heavy metals. Meanwhile, the conservation zone which is relatively protected from industrial activities, shows lower contamination values, although there are still traces of pollution due to remote pollution sources [105], [106].

The PLI value in the industrial area is higher than the conservation zone. This indicates that industrial activities play a role in elevating heavy metal pollution in the area. Industrial areas are usually exposed to pollution sources such as factory waste, air pollution, and surface runoff that carry heavy metals into the sediment [107], [108]. Although both stations are in the same area, there is a difference in the PLI value between Station 1 and Station 2 at both locations. Local factors, including water movement, sediment composition, and proximity to pollution sources, significantly influence the distribution of heavy metals [109]. The PLI in the conservation zone still shows heavy metal pollution. This could be due to atmospheric deposition from industrial activities in the surrounding area or pollutants carried by water currents from more contaminated areas [110], [111]. This suggests that although the conservation zone has better protection, it is not completely protected from the impacts of nearby industrial pollution.

The study indicate that both areas are classified as not polluted. In line with these findings by Karmakar et al. [112], the PLI value in mangrove planting areas due to heavy metals from ship demolition activities is still below 1. Even though the PLI reflects low levels of pollution over time, it can increase the potential for absorption by aquatic organisms and pose ecological risks. therefore, continuous monitoring is required to identify dynamic changes in heavy metal concentrations

### 3.5 Antioxidant non-enzyme activities

The results of percentage of depreciation data for the *A. alba* species taken from the industrial area were 66 %, and the conservation zone was 65.8 %. While for the *E. agallocha* species from the industrial area it was 68.5 % and the conservation zone was 67.9 % in the conservation zone. Conversely, the findings of the percentage of dry weight of *A. alba* in the industrial area were 34 %, and the conservation zone was 34.3 %. In the *E. agallocha*, the percentage of dry weight in the industrial area was recorded at 31.5 % and the zone was 32.1 % (Table 4).

**Table 4:** Depreciation percentage of weight.

Location	Sample leaves	Sample weight (g)		Depreciation percentage (%)	Weight percentage (%)
		Wet	Dry		
Industry area	*A. alba*	800	272	66	34
	*E. agallocha*	800	252	68.5	31.5
Conservation zone	*A. alba*	800	274	65.8	34.3
	*E. agallocha*	800	257	67.9	32.1

The removal of water content from the sample can be achieved by drying it until all moisture is eliminated, as the presence of water can influence the stability of bioactive compounds during extraction. Certain compounds may remain more stable or be less prone to chemical degradation or oxidation in dry conditions. The extraction of leaf samples from *A. alba* and *E. agallocha* was performed using ethanol as the solvent. The results indicated that the extract yield from the *A. alba* leaves was the highest at 8.80 %, which was obtained from the conservation area (Table 5).

**Table 5:** Percentage of etanol extract.

Location	Sample leaves	Extract weight (g)		Depreciation percentage (%)	Extract percentage (%)
		Dry powder	Crude extract		
Industry area	*A. alba*	250	22.01	91.20	8.80
	*E. agallocha*	250	17.33	93.07	6.93
Conservation zone	*A. alba*	250	13.17	94.73	5.27
	*E. agallocha*	250	21.42	91.43	8.57

Based on Table 5, these results indicate that environmental conditions, both in industrial areas and conservation zones, have the potential to affect the weight of crude extracts and the percentage of depreciation of *A. alba* and *E. agallocha* leaves, with the possibility of differences in the composition of bioactive compounds in each location. The maceration and extraction processes are important steps in testing the content of bioactive compounds in samples, especially in separating compound components from mangrove extracts [113]. The use of solvents such as ethanol, which are amphipathic, allows the dissolution of both polar and nonpolar compounds, so that it is optimal for obtaining various bioactive compounds from mangroves, which contain various types of compounds with these properties [113], [114], [115]. A high percentage of extraction weight indicates the effectiveness of the extraction method, indicating the method's ability to obtain active compounds from the sample optimally [116]. High extraction results also indicate a high content of active compounds in the sample, which possess the capability to have biological value and other practical applications [117].

The potential antioxidant content is illustrated by the percentage value of free radical scavenging inhibition along with the IC_50_ value. The results of the antioxidant test on mangrove leaves using the DPPH radical scavenging method using ethanol solvent (Table 6). The IC_50_ value content in the industrial area for *A. alba* of 137.8 μg/ml is classified as a moderate and *E. agallocha* of 21.63 μg/ml is classified as a very strong. While in the conservation area, *A. alba* of 64.32 μg/ml is classified as a strong and *E. agallocha* of 41.43 μg/ml is also classified as a very strong.

**Table 6:** Classification of IC_50_.

Location	Sample leaves	Linear regression			IC_50_ (μg/ml)	Category
		a	b	R^2^		
Industry area	*A. alba*	36.277	128.7	0.9429	137.8	Moderate
	*E. agallocha*	30.953	45.165	0.9419	21.63	Very strong
Conservation zone	*A. alba*	28.726	69.611	0.8905	64.32	Strong
	*E. agallocha*	18.425	18.661	0.904	41.43	Very strong

The IC_50_ classification results indicate that *A. alba* leaves from both areas fall into the strong-moderate category, while *E. agallocha* is classified as very strong. According to Kodikara et al. [118], the difference in the strength of antioxidant activity in each species is thought to be because mangroves have different tolerances to certain environmental conditions, and this can affect the extent to which they can overcome heavy metal toxicity. Previous research explained that the genus *Avicennia* is a mangrove found in the front zone and directly facing the waters [119]. *Avicennia spp.* has strong and dense aerial roots so that it is able to efficiently capture and bind mud and various pollutants carried by water [119], [120]. As a type of plant that is periodically submerged in water, the roots of mangroves are able to take, absorb, or reduce contaminants through the dilution process [121], [122]. Therefore, it is hypothesized that contaminants absorbed by roots do not induce excessive oxidative stress and do not increase the production of secondary metabolites.

Another study in the Island of Weno area, Chuuk State of Micronesia, for the antioxidant activity of *Rhizophora stylosa* roots was 41.3 % and *Sonneratia alba* 40.7 % [61]. While the IC_50_ value of the *E. agallocha* in both areas is included in the high category. *E. agallocha* in this study was found in the ladward zone. This zone is rarely submerged by seawater and is more often affected by lower tides. This is thought to be the cause of the low water content in the leaves of *E. agallocha* as presented in Table 4, so that the pollutants absorbed are greater and last longer in the leaves. Therefore, the roots act to mitigate stress effectively by producing antioxidant activity [123]. The concentration of antioxidant activity (IC_50_) in the leaves showed different values in the two areas. The differences that occur in the ability to produce antioxidant activity in each mangrove as a form of self-defense against oxidative stress are due to differences in morphology, habitat, tides, sediment substrates, and environmental conditions [124], [125]. Kumar et al. [126] also found that mangrove sediments in intertidal zones are rich in organic matter, including phenolic compounds and triterpenoids, which contribute to antioxidant potential. The presence of triterpenoids such as taraxerol acetate, germanicol, and β-amyrin suggests a strong chemotaxonomic link between mangrove-derived organic matter and plant defense mechanisms against oxidative stress. Differences in IC_50_ classification results can reflect differences in the level of heavy metal exposure in the two locations.

In addition to testing antioxidant activity using the DPPH method, this activity can also be analyzed by calculating total phenol. Measuring the total phenol content is done by adding Folin-ciocalteu reagent to the solution sample being tested (Table 7). Phenols possess antioxidant properties that play a role in protecting plant tissues from damage induced by free radicals. Therefore, the total phenol test can provide information about the potential antioxidant activity of mangrove leaf extracts. In this study, the highest quantitative phenol value was found in *E. agallocha* at 398.80 mg GAE/gr from the industrial area and the smallest in *A. alba* at 21.85 mg GAE/gr from the conservation area.

**Table 7:** Total phenol of mangrove leaves extract.

Location	Sample leaves	Phenol (mg GAE/g)
Industry area	*A. alba*	36.68
	*E. agallocha*	398.80
Conservation zone	*A. alba*	21.85
	*E. agallocha*	320.44

The total phenol obtained in this study has a positive relationship with antioxidant activity, as indicated by the IC_50_ value in Table 7. The antioxidant activity of this mangrove is influenced by its total phenol content. The total phenol content is positively correlated with antioxidant activity, where the higher the total phenol content, the higher the antioxidant activity in the sample [66]. Based on this study, *A. alba* has a lower total phenol content than *E. agallocha*, which is strongly suspected due to differences in environmental factors. Mangroves in the pioneer zone more pressure from pollutants and the physicochemical conditions of the habitat. This is in line with previous findings, where the total phenol content in the roots of *A. marina* in the pioneer zone was 26.11 mg GAE/g, lower than *B. gymnorrhiza* in the landward zone with 344.02 mg GAE/g [127]. Mangrove ecosystems located in the pioneer zone tend to have special adaptations to survive in coastal environments that are often inundated by sea tides [128], [129]. Mangrove sediments in intertidal zones are rich in organic matter originating from terrestrial vascular plants, including phenolic compounds and triterpenoids, which contribute to their antioxidant potential [126]. Mangroves mitigate pollutants by reducing their concentration and toxicity through internal water content regulation, preventing excessive accumulation of absorbed contaminants [130]. According to Laoué et al. [131], non-enzymatic antioxidant activity is not produced exclusively because there is a certain limit for excess free radicals. However, the non-enzymatic antioxidant system is usually activated when free radical levels or oxidative stress exceed normal defense capacity [132].

**Fig. 5:**
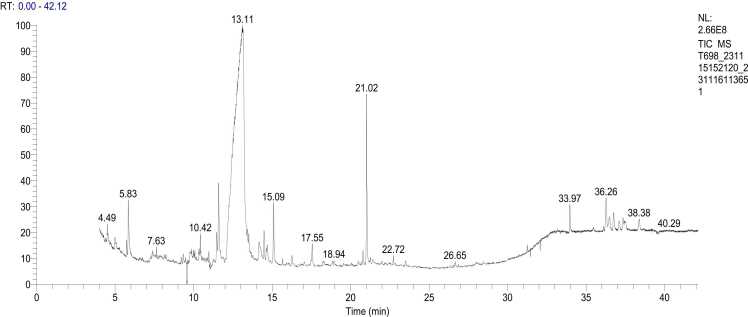
GC-MS chromatogram of bioactive compounds in mangrove leaves *E. agallocha* (Industry area).

GC-MS analysis using *E. agallocha* mangrove leaf samples from industrial areas because they are included in the IC_50_ classification is very strong among others. The graph revealed 15 peak points identifying compounds such as flavonoids, lipids, glycosides, glucosinolates, glucose, esters, terpenoids, and fatty acids (Fig. 5). The identified compounds, based on chromatogram peak heights and mass spectra from the analysis, match those in the WILEY 7 database library (Table 8).

**Table 8:** Retetion time, peak area, compound name, formula, and compound group (*E. agallocha*).

*Ret. time*	*Peak Area %*	Compound name	Formula	Compound group
5.84	2.45	4H-Pyran−4-one, 2,3-dihydro−3,5-dihydroxy−6-methyl	C_6_H_8_O_4_	Flavonoid
9.49	1.68	2-Myristynoyl pantetheine	C_23_H_45_N_2_O_4_S	Lipid
9.77	1.65	Paromomycin	C_23_H_45_N_5_O_14_	Glikosida
9.87	1.17	2-Myristynoyl pantetheine	C_23_H_45_N_2_O_4_S	Lipid
11.46	1.16	Desulphosinigrin	C_11_H_21_NO_9_S_2_	Glukosinolat
11.59	3.31	2-O-Methyl-D-mannopyranosa	C_7_H_14_O_6_	Glikosida
13.10	73.97	3-O-Methyl-d-glucose	C_7_H_14_O_6_	Glukosa
14.16	1.84	3-O-Methyl-d-glucose	C_7_H_14_O_6_	Glukosa
14.48	0.99	7-Methyl-Z-tetradecen−1-ol acetate	C_17_H_34_O_2_	Ester
14.69	1.05	9-Octadecenoic acid, (2-phenyl−1,3-dioxolan−4-yl)methyl ester, trans-	C_28_H_44_O_4_	Ester
15.09	2.29	2,6,8-Trimethylbicyclo[4.2.0]oct−2-ene −1,8-diol	C_11_H_18_O_2_	Terpenoid
17.55	0.98	Hexadecanoic acid, methyl ester	C_17_H_34_O_2_	Asam lemak
21.01	4.87	Phytol	C_20_H_40_O	Terpenoid
33.97	0.94	9-Desoxo−9-x-acetoxy−3,8,12-tri-O-ac etylingol	C_21_H_30_O_9_	Glikosida
36.27	1.65	1-Monolinoleoylglycerol trimethylsilyl ether	C_21_ H_44_ O_4_ Si	Ester

Based on Table 8, 8 groups of compounds were found. The groups of compounds that are thought to be formed in response to the environment that increases antioxidant activity, such as flavonoids, lipids, glycosides, glucosinolates, glucose, esters, terpenoids, and fatty acids. The compound 4H-Pyran-4-one, 2,3-dihydro-3,5-dihydroxy-6-methyl found in these leaves is classified as a flavonoid. Flavonoids are specialized metabolites commonly found in plants, serving multiple functions such as defense and signaling, particularly under stress conditions [131]. Flavonoids are categorized into several groups, including chalcones, aurones, flavanonols, flavones, isoflavones, flavanols, flavonols, anthocyanins, proanthocyanidins, and leucoanthocyanidins. They can exist as aglycones, glycosides, and methylated derivatives. The compounds 2-Myristynoyl pantetheine and 2-O-Methyl-D-mannopyranose are classified as lipids. Lipid compounds can exhibit antioxidant activity, especially through mechanisms involving phenols and other structures that modulate oxidative stress and lipid peroxidation processes [133].

The compounds Paromomycin, 2-O-Methyl-D-mannopyranose, and 9-Desoxo-9-x-acetoxy-3,8,12-tri-O-ac etylingol are classified as glycoside compounds. Based on the results of the study of Yang et al. [134], flavonoid glycosides are widely distributed in plants, where they function as phytoalexins to combat biotic stress. Desulphosinigrin is a glucosinolate known to exhibit anticancer and antimicrobial properties [135]. 4-methylsulfinylbutyl glucosinolate is a glucosinolate derived from the amino acid methionine, which has antioxidant, antifungal, and antimicrobial activities [136]. The compounds 7-Methyl-Z-tetradecen-1-ol acetate, 9-Octadecenoic acid, (2-phenyl-1,3-dioxolan-4-yl) methyl ester, trans-, and 1-Monolinoleoylglycerol trimethylsilyl ether are classified as esters. Clearly show that ester groups with different aromatic and alkyl chains will increase antioxidant capacity. e compound 2-[4-methyl-6-(2,6,6-trimethylcyclohex-1-enyl)hexa-1,3,5-trienyl]cyclohex-1-en-1 carboxaldehyde is categorized as an aldehyde. This type of compound is commonly found in various essential oils and contributes a distinctive aroma to certain plants. Several phenolic aldehydes and derivatives have antioxidant activity [137].

Compounds 2,6,8-Trimethylbicyclo[4.2.0]oct-2-ene −1,8-diol and phytol belong to the terpenoid compound group. Terpenoids are promising lead compounds for further structural modification and optimization because of their potent anti-inflammatory effects [138], [139]. Terpenoids (such as monoterpenes and carotenoids) and polyphenols (such as quercetin and other flavonoids) are important phytochemicals with various antioxidant effects [140]. Hexadecanoic acid, methyl ester compounds are classified as fatty acid compounds. Fatty acids have been found to be associated with various biological activities such as anti-inflammatory, antioxidant, antifeedant, antimicrobial, and neuroprotective [141]. While compounds that have no relationship with antioxidant activity are the glucose compound group found in leaf extracts. Glucose produced through photosynthesis and other carbohydrate processes can be used as an energy source to maintain cell vitality [142].

### 3.6 Correlation of heavy metal concentrations and biomarkers

The relationship between heavy metal concentrations and antioxidant activities in mangrove leaves in both areas using Pearson correlation analysis, which begins with assumption testing (Table 9). The test results were obtained for all variables with significance > 0.05, and if the skewness and quasi-sequence ratios are in the range of −1.96 and + 1.96, it can be concluded that the data distribution is normal.

**Table 9:** Assumption test results.

Sample	Variable	Mean	St.Dev	Sig.2 tailed	Skewness Kurtosis	Values
Leaves	Pb	0.94	0.12	0.927	0.55 dan 0.55	Normal
	Cu	3.57	0.080	0.498	0.33 dan 1.35	Normal
	IC50	66.35	25.19	0.457	1.31 dan 0.69	Normal
	Total Phenol	194.44	193.48	0.182	0.13 dan 1.93	Normal

Based on the results of the assumption test, the normal distribution of the data can explain that the statistical parameters used in the correlation analysis provide an accurate picture of the center and distribution of the data. Furthermore, the results of the pearson correlation test (r) and the coefficient of determination (Kd) are summarized in Table 10.

**Table 10:** Results of the Pearson correlation test (r) and coefficient of determination (Kd).

Sample	Variable (X-Y)	r	Kd (%)	Interpretation
Leaves	Pb – IC_50_	−0.906	82.08	Strong correlation
	Cu – IC_50_	−0.937	87.79	Strong correlation
	Pb – Total Phenol	0.904	81.72	Strong correlation
	Cu – Total Phenol	0.949	90.06	Strong correlation

The results of the correlation test is a significant correlation or relationship between heavy metals and physiological responses (r ≠ 0). The relationship between Pb and Cu to antioxidant activity in mangrove leaves produced from both areas has a very high negative correlation direction of −0.906 and −0.937. The relationship between Pb and Cu to total phenol in leaf samples is also very strong, with a very high positive correlation value of 0.904 and 0.949. In addition, the percentage of the determination coefficient (Kd) indicates that variables X and Y have a strong relationship. The Kd value of mangrove leaf samples ranges from 81.72 % to 90.06 %. This indicates that most of the variations in IC_50_ and total phenol can be explained by the Pb and Cu variables in both types of samples.

A high correlation indicates a strong relationship between the variables concerned and significantly supports the hypothesis. A negative relationship with IC_50_ indicates that the higher the concentration of Pb or Cu, the lower the IC_50_ value (higher antioxidant potential). A positive relationship with total phenol indicates that the higher the concentration of Pb or Cu, the total phenol content also increases. Furthermore, the results of GCMS screening also showed the presence of compounds such as flavonoids, lipids, glycosides, glucosinolates, glucose, esters, terpenoids, and fatty acids. Previous studies have shown that some of these compounds, especially the flavonoid and terpenoid groups, have significant antioxidant activity [143]. Therefore, increasing concentrations of heavy metals can indirectly affect the profile of secondary metabolite compounds in mangrove plants, which in turn can affect antioxidant activity and response to oxidative stress. Excessive concentrations of heavy metals cause the formation of ROS and affect the activity of antioxidants involved in plant metabolism [144]. According to Georgiadou et al. [145], detoxification of ROS due to heavy metal contamination by producing antioxidant enzymes plays a central and vital role in protection in mangrove species.

In line with the research by [146], that under abiotic stress conditions, such as heavy metal contamination, the production of reactive oxygen species (ROS) increases in plants, resulting in the induction of oxidative stress, and plants initiate antioxidant production that significantly delays or prevents oxidative stress. Secondary metabolite compounds are involved in plant responses to biotic and abiotic stresses and contribute significantly to the antioxidant activity of plant tissues [103]. Antioxidant activity is a common approach used to increase heavy metal tolerance, strengthening the defense system against oxidative stress [146], [147]. Several previous studies have found a relationship between heavy metal pollution and the physiological response of plants, especially mangroves. The decline in sediment quality due to heavy metal pollution in a gradual pattern that has the potential to have a negative impact on the biogeochemical cycle, with potentially fatal consequences for the survival of biodiversity (*A. marina*) [148]. Furthermore, the results of the study by Ghosh et al. [149] also stated that there was a statistically significant relationship between the activity of antioxidant enzymes, photosynthetic pigments, and heavy metal contamination, resulting in the biotic response of riparian mangroves characterized by reduced photosynthetic pigments (chlorophyll a and b) and increased activity of antioxidant stress enzymes (POD, CAT, and SOD). The response of two tropical medicinal plant species to heavy metal accumulation can increase hydrogen peroxide (H_2_O_2_) activity, malondialdehyde content, enzymatic activity, and nonenzymatic antioxidants [149].

Mangroves cause trigger antioxidant defenses to overcome heavy metal absorption and normalize excessive production of oxidative stress mediated by reactive oxygen species (ROS) [150]. However, antioxidant responses in mangroves vary depending on the concentration and type of heavy metals, plant species, and duration of exposure [151]. Previous findings related to plant reactions to higher concentrations of heavy metals in the soil. For example, Kulbat-Warycha et al. [152] observed that an increase in the concentration of heavy metals (Ni, Cu, Zn) caused a decrease in the concentration of phenols in oregano, which was associated with the induction of severe oxidative stress. According to Mansoor et al. [153], excessive ROS production due to severe oxidative stress can cause damage to the mitochondrial respiratory chain, uncoupling of oxidative phosphorylation, and mitochondrial death in plants. However, this can also experience a decrease in the antioxidant activity defense system of the mangrove itself if the contamination of absorbed pollutants exceeds the threshold and severe oxidative stress occurs, which can cause damage and death to the mangrove ecosystem [154], [155].

The correlation between heavy metals and antioxidant activity in mangroves illustrates the complex relationship between heavy metal pollution and plant responses to oxidative stress. In this context, high concentrations of heavy metals can trigger ROS production, which in turn affects plant antioxidant activity. Excessive ROS can induce oxidative stress that activates the plant defense system to increase the production of antioxidant compounds. Thus, the relationship between heavy metals and antioxidant activity, total phenols, and secondary metabolite compound profiles in mangroves provides a deeper understanding of the mechanism of the plant's response to heavy metal pollution and oxidative stress. Therefore, if there is an indication that pollutant contamination exceeds the threshold and causes severe oxidative stress, some coastal environmental management policies can be expected in response to these findings.

To ensure the sustainability of mangrove ecosystems and mitigate the impact of heavy metal pollution, routine monitoring is recommended every 3–6 months to capture seasonal variations in heavy metal concentrations and antioxidant responses. Additionally, long-term monitoring (≥5 years) is necessary to identify trends in heavy metal accumulation and its effects on coastal ecosystems. Supplemental monitoring is also advised following specific events, such as industrial waste spills or land-use changes, to assess their immediate environmental impact. The data from this study can serve as a basis for environmental policy development, including updating regulations on heavy metal thresholds in sediments and coastal biota, strengthening conservation and mangrove rehabilitation policies, and improving industrial zone management in coastal areas. Furthermore, these findings can be utilized to raise public awareness about the importance of protecting coastal ecosystems and promoting sustainable resource management practices.

## 4 Conclusion

Heavy metal pollution of Pb and Cu resulting from areas affecting industrial and conservation activities has a significant effect on antioxidant activity in mangroves (*A. alba* and *E. agallocha*). Sediment pollution assessment showed that the Igeo value was at a low level, while the contamination factor (Cf) and pollution load Index (PLI) showed a relatively moderate level of pollution (Cf between 1 and 3, and PLI between 0 and 2). The bioaccumulation value of heavy metals in mangrove leaves was low (BCF < 1), indicating moderate accumulation of heavy metals in leaf tissue. The antioxidant activity of *E. agallocha* leaves from the industrial area was very strong and had the highest total phenol content. The compounds identified as having high antioxidant activity included flavonoids, lipids, glycosides, glucosinolates, glucose, esters, terpenoids, and fatty acids. Correlation analysis showed that increasing heavy metal concentrations were directly proportional to increasing antioxidant activity and total phenol content in mangrove leaves. This study contributes to our understanding of the potential of mangroves to respond to heavy metal exposure through increased antioxidant activity, which can support conservation efforts and sustainable management of coastal natural resources.

## Author statement

The authors hereby declare that the work presented in this article is original and that any liability for claims relating to the content of this article will be borne by them.

## CRediT authorship contribution statement

**Rozirwan:** Writing – review & editing, Supervision, Project administration, Funding acquisition, Conceptualization. **Nadila Nur Khotimah:** Writing – original draft, Validation, Resources, Formal analysis, Data curation. **Wike Ayu Eka Putri:** Software, Investigation. **Fauziyah:** Supervision, Data curation. **Riris Aryawati:** Methodology, Data curation. **Gusti Diansyah:** Software, Investigation. **Redho Yoga Nugroho:** Writing – review & editing, Resources, Formal analysis, Data curation.

## Declaration of Competing Interest

The authors declare that they have no known competing financial interests or personal relationships that could have appeared to influence the work reported in this paper.

## Data Availability

No data was used for the research described in the article. The data supporting the findings of this study can be obtained from the corresponding author upon a reasonable request.
